# Robotic Single-Site Surgery for Female-to-Male Transsexuals: Preliminary Experience

**DOI:** 10.1155/2014/674579

**Published:** 2014-04-10

**Authors:** Stefano Bogliolo, Chiara Cassani, Luciana Babilonti, Barbara Gardella, Francesca Zanellini, Mattia Dominoni, Valentina Santamaria, Rossella Elena Nappi, Arsenio Spinillo

**Affiliations:** Department of Obstetrics and Gynaecology, IRCCS-Fondazione Policlinico San Matteo and University of Pavia, 19 Viale Camillo Golgi, 27100 Pavia, Italy

## Abstract

Hysterectomy with bilateral salpingo-oophorectomy is a part of gender reassignment surgery for the treatment of female-to-male transsexualism. Over the last years many efforts were made in order to reduce invasiveness of laparoscopic and robotic surgery such as the introduction of single-site approach. We report our preliminary experience on single-site robotic hysterectomy for cross-sex reassignment surgery. Our data suggest that single-site robotic hysterectomy is feasible and safe in female-to-male transsexualism with some benefits in terms of postoperative pain and aesthetic results.

## 1. Introduction 

Transsexualism is a condition in which a person lives a significant incongruence between gender identity and physical phenotype [1]. This conflict causes general suffering called gender dysphoria and a strong desire to live and be accepted as a member of the opposite sex. Treatment for person with gender identity disorder is based on psychiatric and psychological support, hormonal therapy, and gender reassignment surgery [1]. Female-to-male reassignment surgery is a series of complex surgical procedures which may include mastectomy, hysterectomy with bilateral salpingo-oophorectomy (BSO), and penile and scrotal reconstruction. The aim of our study is to evaluate if robotic single-site hysterectomy with BSO could play a role in reassignment surgery for female-to-male transsexualism (FMT).

## 2. Material and Methods

We conducted a retrospective analysis of perioperative data from ten consecutive patients who underwent robotic-assisted single-site laparoscopic hysterectomy (RSSH) for FMT at our institution from April to December 2013. Candidates for gender reassignment were referred to us for the operation from various centers. All patients had diagnosis of gender identity disorder assessed by mental health professional according to DSM-IV-TR criteria [2] and all of them met World Professional Association for Transgender Health Standards [3] including documentation of long term desire of transition to the other sex, living as a male, and receiving testosterone for at least six months. Inclusion criteria in all cases were age > 18 years and absence of any contraindication to endoscopic surgery. After Institutional Ethics Committee approval, specific written informed consent was taken from all the patients in agreement with local and international legislation (Declaration of Helsinki). Participants were also informed that the RSSH may be converted to laparoscopic or laparotomic hysterectomy, in presence of any specific difficulties.

Data regarding baseline patients' characteristics (age, BMI, previous surgery, comorbidities, smoke, hormonal therapy, parity, and sexual function) were collected as reported in Table 1.

Visual Analogic Scale ranging from 0 = no pain to 10 = agonizing pain was used to evaluate postoperative pain, and VAS score was recorded every 3–6 hours for all patients from the end of surgery till discharge. According to our anesthesia protocol standard analgesic therapy with ketorolac 30 mg twice a day and acetaminophen 1000 mg every 8 hours was administered, while tramadol was used only on demand. At the end of surgery ropivacaine local infiltration was performed at the single-site port access.

All patients received venous thromboembolism and antibiotic prophylaxis according to institutions guidelines.

Every surgery was performed using a da Vinci Si Surgical System (Intuitive Surgical, Sunnyvale, CA, USA) by a team consisting of two experienced surgeons (Stefano Bogliolo and Luciana Babilonti) and one bedside assistant for uterine manipulation. The single-site system is a multichannel port device not reusable with space for 4 cannulae and an insufflation valve. The specific cannulae are as follows: two 250 mm in length curved cannulae for robotic instruments, one cannula for the high-definition three-dimensional endoscope, and one 5 or 10 mm assistant surgeon cannula.

### 2.1. Surgical Technique

**Figure 1:**
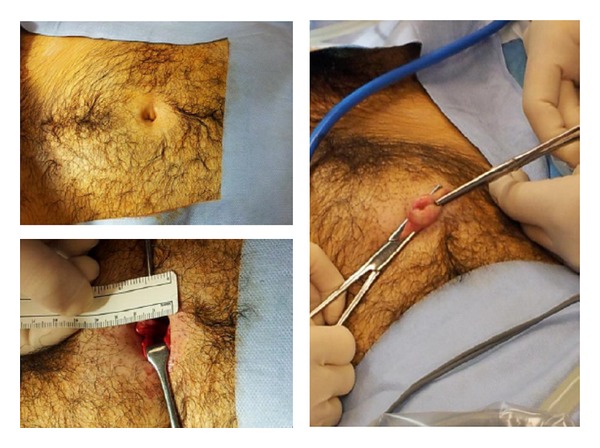
Single-site umbilical incision.

Uterine manipulator device was placed, when possible, in order to improve uterine mobilization. A 2 cm incision was performed at the umbilical scar in correspondence with the physiological hernia (Figure 1) and single-site port, previous lubrication with a sterile saline solution, was introduced in abdominal cavity within a descending movement.

Pneumoperitoneum up to 12 mmHg of pressure was started and patient was placed in lithotomy position at the 30° Trendelenburg position. da Vinci Si robotic column was positioned between the patient's legs and da Vinci Si 8.5 mm 30° endoscope was placed in umbilical trocar. After that, two 5 × 250 mm curved cannulae were introduced through the specific lumen, under constant visualisation and eventually the specific robotic instruments were placed (surgical forceps on Arm 2 and curved scissor on Arm 1). The assistant's 5 or 10 mm cannula was also inserted allowing the use of classic bipolar instruments overcoming the absence of specific robotic one.

The technique used for robotic single-site hysterectomy and BSO was similar to that used in standard laparoscopy; uterus and adnexa were removed through the vagina and vaginal cuff was closed both vaginally and robotically depending on anatomic features.

## 3. Results and Discussion 

A series of 10 patients underwent RSSH + BSO at our department during nine months. The clinic-pathological characteristics of these patients were reported in Table 1. The median patients' age was 28 ± 5.7 years (range 20–40) and the median BMI was 22 ± 1.7 kg/m^2^ (range 19–25).

Regarding surgical procedure in all cases a total hysterectomy with BSO was performed.

The mean operative time was 137 ± 32 min (range 90–210), the mean console time was 79 ± 15 min (range 55–110), and the mean docking time was 9 ± 2 min (range 6–18). The mean intraoperative blood loss was 30 ± 24 mL (range 15–100). The median uterine weight was 89 ± 15 gr (range 60–120).

The vaginal cuff closure was performed in eight cases transvaginally; only in two cases of virgin patients the vaginal suture was performed with extracorporeal knots, with the aid of a push knot, as previously described [4].

Regarding perioperative outcomes, no laparoscopic or laparotomic conversion was needed during robotic surgical procedure.

Only in one case a minor postoperative complication occurred: vaginal bleeding required a partial vaginal suture for hymeneal ring laceration, due to important atrophy.

The median hospital stay was 2.4 ± 0.9 days (range 2–5). All patients underwent the same surgical analgesic protocol without opioids and they experienced low postoperative pain (VAS value: 1 IQR 0–3 at 60 minutes and VAS value: 0 IQR 0-0 at 24 hours after intervention).

### 3.1. Discussion

Traditionally, in sex reassignment surgery, total laparoscopic hysterectomy with BSO is performed. Vaginal route, ideally “scarless” surgery, cannot routinely be offered to FMT patients because most transsexuals are nulliparous and virginal and have narrow and atrophic vaginal walls as a result of long term hormonal therapies [5]. But even if they are small, classic laparoscopic incisions, one suprapubic and two lateral symmetrically placed over the anterior superior ileac crests, could be easily recognized as a “mark” of gynaecologic surgery. In recent years the phenomenon of endoscopic single-site surgery started a new way in reducing invasiveness of both laparoscopic and robotic surgery in gynecology, achieving the goal of reduction of postoperative pain and improving patients' cosmetic satisfaction [6–10]. Furthermore robotic-assisted single-site approach seems to overcome some technical limitations of laparoendoscopic single-site surgery due to increased movements of instruments and ergonomics [11, 12].

At present there are no experiences describing robotic single-site technique in FMT; therefore a comparison between our preliminary data and perioperative outcome of similar reports is not possible.

Indeed the surgical technique of single-site hysterectomy is not different from that used in benign gynaecological disease and our FMT data are in line with those reported in literature for female patients [11, 13]. Nevertheless we recognize that this study has limitations, which include the retrospective design and the small number of patients.

Cross-sex surgery should be ideally offered to all transsexuals who do not desire fertility, for different reasons. First, according to literature, surgical reassignment allows amelioration of quality of life in several meaningful areas (socioprofessional, relationship, and psychological) and improves social and sexual functioning [14]. Moreover the ablative genital surgery can reduce the risk of hormone-dependent cancers related to long term testosterone exposure [15].

At present only few studies focused on surgical outcome and technique of hysterectomy with BSO for female-to-male transsexualism. In 1999 Ergeneli et al. first reported the feasibility and safety of laparoscopically assisted vaginal hysterectomy in FMT reassignment surgery in eight patients who subsequently underwent phallic construction. According to the author laparoscopy was useful allowing preservation of structures necessary for phallic reconstruction [16].

In 2007 O'Hanlan et al. reported the largest series in literature evaluating surgical outcome of 41 transsexual patients compared to normal female patients who underwent total laparoscopic hysterectomy and BSO [5]. The authors concluded that there were not significant differences in complications and no complications sustained by the transsexuals were unique to their status.

Recently in 2013 Lazard et al. reported a series of ten patients who underwent single-point access laparoscopic hysterectomy for sex reassignment with no conversion to standard laparoscopy or laparotomy [17].

Similar to Lazard, in our peculiar series, robotic single-site approach allowed performing total hysterectomy and BSO with 100% success. The use of single intraumbilical incision made scar completely hidden; thus transsexual patients did not bear the “stigma” of standard laparoscopic gynecologic surgery [18]. Moreover the ultimate goal of reassignment surgery is a functional phallus; thus in excising generative organs gynecologists are asked to preserve structures vital for subsequent reconstructive surgery. Single transumbilical incision avoids the risk of interrupting the inferior epigastric or circumflex ileac circulation, potentially important for future genital reassignment surgery.

Robotic single-site surgery in our experience achieved the goal of excellent postoperative pain control as already highlighted by other authors [8, 12]. All patients did not require supplementary drugs for pain control. Ropivacaine wound infiltration was performed at the end of operation according to literature data [19], in minimizing pain response to surgical trauma.

General satisfaction about surgery and aesthetic results are objective of an ongoing study at our institution. However preliminary data regarding the first five patients at 1–3 and 6 months reported a satisfaction VAS score >8 for both aspects.

## 4. Conclusion 

Larger series and long term follow-up data are still lacking, but we consider single-site robotic surgery a valid choice in FMT reassignment surgery, with a low rate of complications, good pain control, and excellent aesthetic results. Indeed in our small series only a minor perioperative complication occurred and not directly related to single-site technique.

## Figures and Tables

**Table 1:** Patients' characteristics.

Characteristics	Values
Age (years)	28 ± 5.7 (M ± DS)
BMI (kg/m^2^)	22 ± 1.7 (M ± DS)
Parity	—
Previous abdominal surgery	4 (40%)
Mastectomy	9 (90%)
Comorbidities	2 (20%)
Smoke	10 (100%)
Androgen therapy (years)	2.6 ± 1.2 (M ± DS)
Virgin	2 (20%)

## References

[1] Gooren LJ (2011). Care of transsexual persons. *The New England Journal of Medicine*.

[2] American Psychiatric Association (2000). *Diagnostic and Statistical Manual of Mental Disorders*.

[3] Coleman E, Bockting W, Botzer M (2012). Standards of care for health of transsexual, transgender, and gender-nonconforming people, version 7. *International Journal of Transgederism*.

[4] Bogliolo S, Cassani C, Babilonti L, Spinillo A (2013). Vaginal cuff closure during robotic single-port hysterectomy: is vaginal route always the best one?. *Surgical Endoscopy*.

[5] O’Hanlan KA, Dibble SL, Young-Spint M (2007). Total laparoscopic hysterectomy for female-to-male transsexuals. *Obstetrics & Gynecology*.

[6] Fanfani F, Fagotti A, Scambia G (2010). Laparoendoscopic single-site surgery for total hysterectomy. *International Journal of Gynecology and Obstetrics*.

[7] Fanfani F, Rossitto C, Gagliardi ML (2012). Total laparoendoscopic single-site surgery (LESS) hysterectomy in low-risk early endometrial cancer: a pilot study. *Surgical Endoscopy*.

[8] Fagotti A, Bottoni C, Vizzielli G (2011). Postoperative pain after conventional laparoscopy and laparoendoscopic single site surgery (LESS) for benign adnexal disease: a randomized trial. *Fertility and Sterility*.

[9] Escobar PF, Bedaiwy MA, Fader AN, Falcone T (2010). Laparoendoscopic single-site (LESS) surgery in patients with benign adnexal disease. *Fertility and Sterility*.

[10] Mereu L, Pontis A, Carri G, Mencaglia L (2011). Single-port access laparoscopic hysterectomy: a new dimension of minimally invasive surgery. *Journal of Gynecological Endoscopy and Surgery*.

[11] Cela V, Freschi L, Simi G, Ruggiero M, Tana R, Pluchino N (2013). Robotic single-site hysterectomy: feasibility, learning curve and surgical outcome. *Surgical Endoscopy*.

[12] Vizza E, Corrado G, Mancini E (2013). Robotic single-site hysterectomy in low risk endometrial cancer: a pilot study. *Annals of Surgical Oncology*.

[13] Sendağ F, Akdemir A, Oztekin MK (2013). Robotic single-incision transumbilical total hysterectomy using a single-site robotic platform: initial report and technique. *The Journal of Minimally Invasive Gynecology*.

[14] Parola N, Bonierbale M, Lemaire A, Aghababian V, Michel A, Lançon C (2010). Study of quality of life for transsexuals after hormonal and surgical reassignment. *Sexologies*.

[15] Mueller A, Gooren L (2008). Hormone-related tumors in transsexuals receiving treatment with cross-sex hormones. *European Journal of Endocrinology*.

[16] Ergeneli MH, Duran EH, Özcan G, Erdogan M (1999). Vaginectomy and laparoscopically assisted vaginal hysterectomy as adjunctive surgery for female-to-male transsexual reassignment: preliminary report. *European Journal of Obstetrics Gynecology and Reproductive Biology*.

[17] Lazard A, Cravello L, Poizac S, Gorin-Lazard A, Gamerre M, Agostini A (2013). Hysterectomy and bilateral adnexectomy by laparoscopic single port access for female to male transsexualism. *The Journal of Sexual Medicine*.

[18] Bogliolo S, Cassani C, Babilonti L, Musacchi V, Nappi RE, Spinillo A (2014). Robotic single site hysterectomy with bilateral salpingo-oophorectomy in female to male transsexualism. *The Journal of Sexual Medicine*.

[19] Joshi GP, Bonnet F, Kehlet H (2013). Evidence-based postoperative pain management after laparoscopic colorectal surgery. *Colorectal Disease*.

